# Supplementary material for: DMRforPairs: identifying Differentially Methylated Regions between unique samples using array based methylation profiles
Source: BMC Bioinformatics. 2014 May 15;15:141. doi: 10.1186/1471-2105-15-141 (PMC4046028; doi:10.1186/1471-2105-15-141)
Supplement: Additional file 2 — DMRforPairs output for the comparison of A431-MCF7 and NA17018-NA17105. Please start from the HTML files in each folder. Available via the BMC Bioinformatics website. [file 1471-2105-15-141-S2.zip › 1394847754114233_MOESM2_ESM/A431_MCF7/figures/11562.pdf]

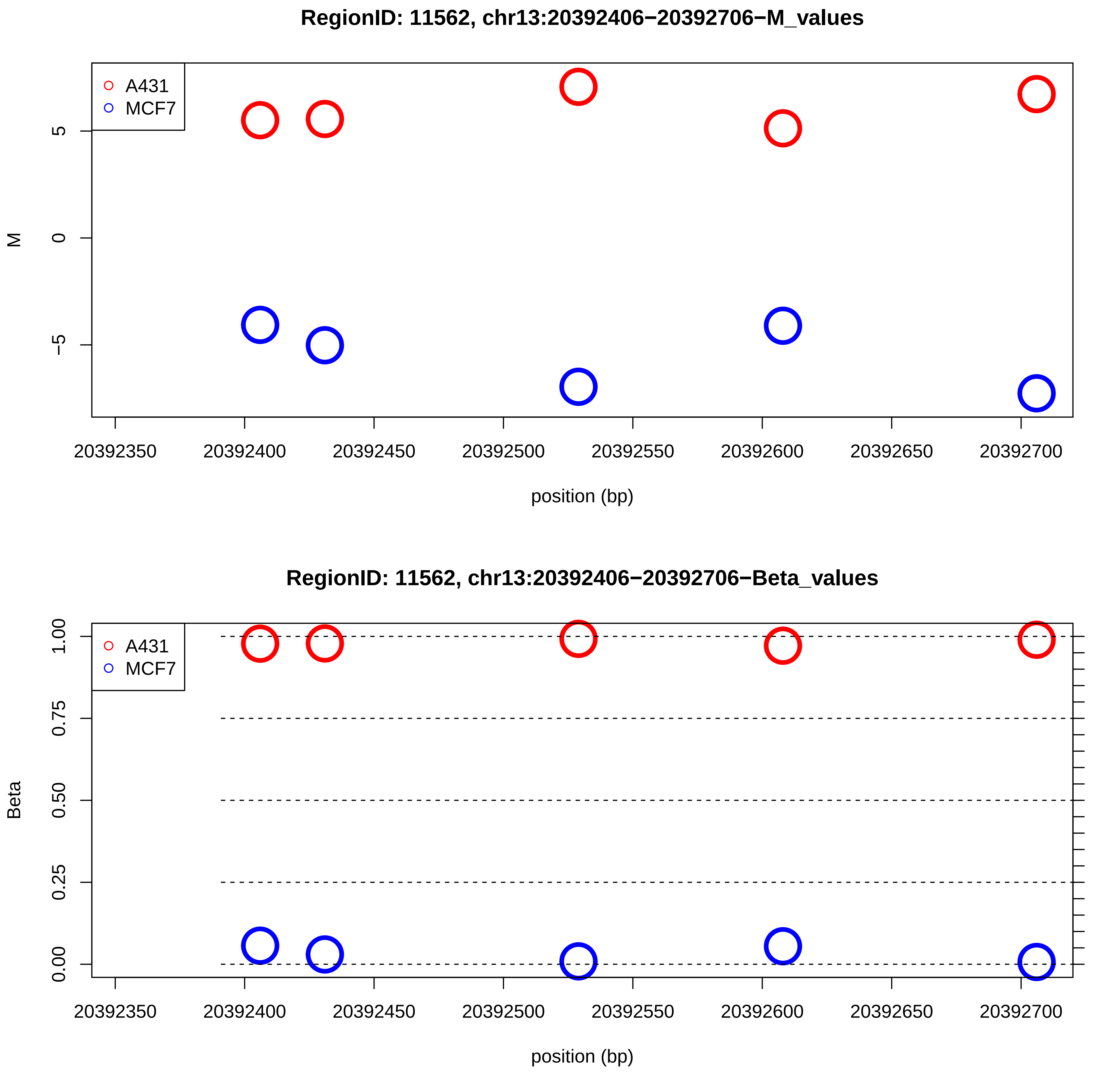
RegionID: 11562, chr13:20392406−20392706−M_values
A431
MCF7
M
5
0
−5
20392350
20392400
20392450
20392500
20392550
20392600
20392650
20392700
position (bp)
RegionID: 11562, chr13:20392406−20392706−Beta_values
A431
MCF7
Beta
1.00
0.75
0.50
0.25
0.00
20392350
20392400
20392450
20392500
20392550
20392600
20392650
20392700
position (bp)

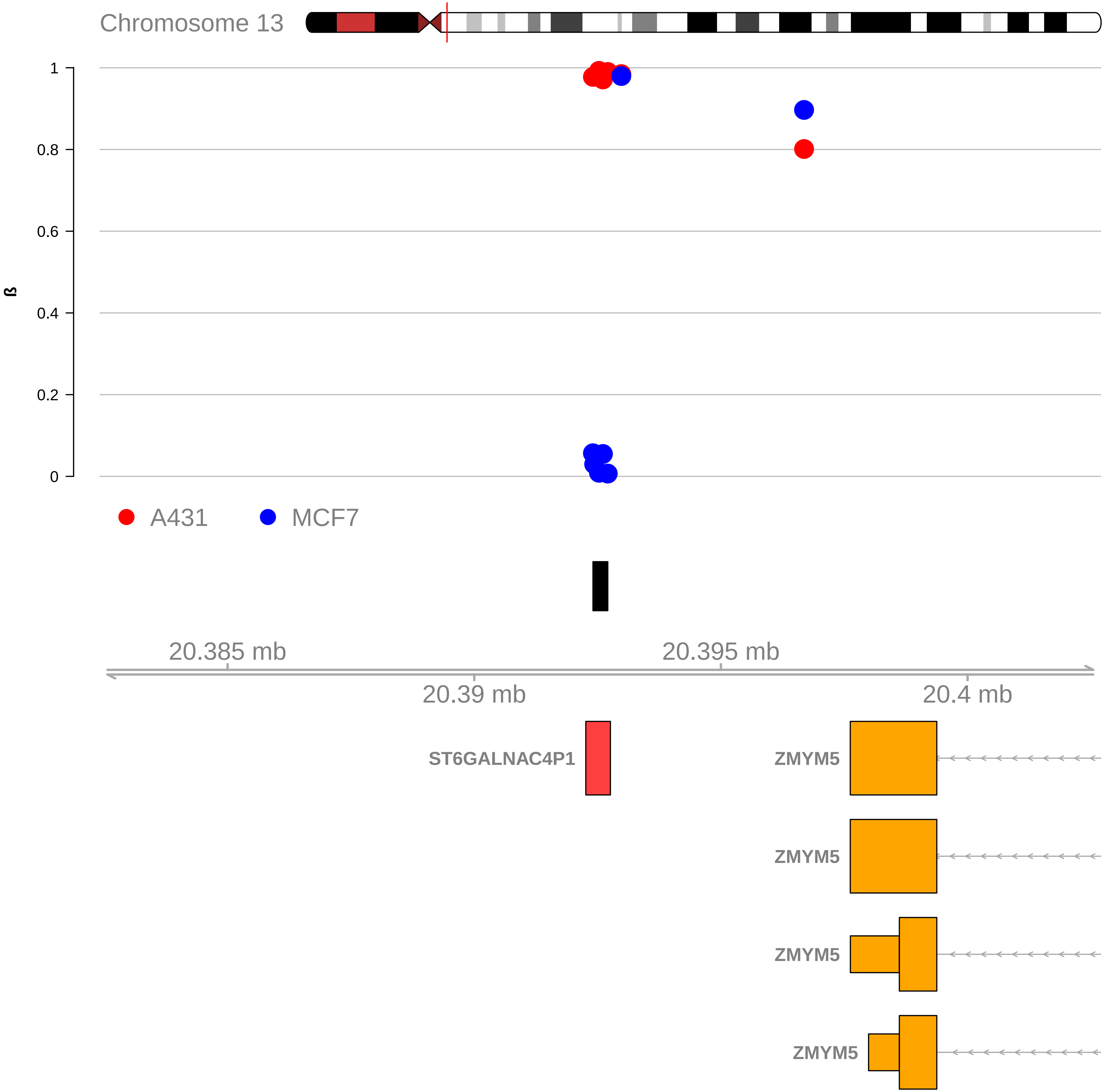

Chromosome 13
ß
1
0.8
0.6
0.4
0.2
0
A431
MCF7
20.385 mb
20.395 mb
20.39 mb
20.4 mb
ST6GALNAC4P1
ZMYM5
ZMYM5
ZMYM5
ZMYM5
